# Thermal Burn of the Larynx in a Child Caused by Microwaved Food

**DOI:** 10.7759/cureus.99610

**Published:** 2025-12-19

**Authors:** Kensuke Kawamoto, Susumu Sato, Akihiro Kaki, Tatsuro Nishi, Takafumi Yamano

**Affiliations:** 1 Department of Otorhinolaryngology-Head and Neck Surgery, Fukuoka University, Fukuoka, JPN; 2 Section of Otorhinolaryngology, Department of Medicine, Fukuoka Dental College, Fukuoka, JPN

**Keywords:** airway obstruction, edema and obstruction of the airway, laryngeal injury, microwave oven, thermal burn

## Abstract

Thermal burns of the larynx are uncommon complications of ingesting overheated food and can lead to life-threatening upper airway obstruction due to edema. Here, we report the case of a 10-year-old boy who developed throat pain shortly after taking a microwave-heated rice ball. The following day, he presented to an emergency medical facility because his symptoms persisted and worsened. Although his vital signs and respiratory status were stable with minimal oral findings, flexible laryngoscopy demonstrated marked supraglottic edema consistent with thermal burn injury. Due to the risk of delayed airway obstruction, endotracheal intubation was performed under multidisciplinary support, and he was admitted to the intensive care unit. Intravenous corticosteroids and antibiotics were administered, resulting in a gradual reduction in laryngeal edema. He was successfully extubated on hospital day 4 and discharged without any complications. This case highlights that microwave-heated food can cause laryngeal thermal injury and emphasizes the importance of early endoscopic evaluation and proactive airway management when upper airway involvement is suspected.

## Introduction

Thermal burns of the larynx are rare, as overheated food or beverages are usually expelled before the thermal damage extends to the larynx. Only a limited number of cases have been reported, some of which occurred in children, and only one case has been associated with solid foods [1,2]. Microwave-heated foods pose a unique risk because heterogeneous heating can generate localized "hot spots" that may cause direct laryngeal injury without visible oral burns [3-5].

Although the management of laryngeal thermal injury is generally established, awareness of this preventable yet potentially life-threatening condition remains limited, particularly among pediatric populations.

This case highlights an underrecognized mechanism of injury, laryngeal thermal burns caused by microwave-heated solid food, and emphasizes the importance of early recognition and airway management to prevent airway obstruction.

Because thermal burns of the larynx may progress with delayed edema and cause airway compromise, early diagnosis and appropriate management are crucial. Here, we report a rare pediatric case of microwave-heated food and describe its clinical course.

## Case presentation

The patient was a previously healthy 10-year-old boy who consumed a rice ball heated in a microwave oven the evening before admission. Owing to the high temperature, he immediately spat it out but subsequently developed throat pain and dysphagia. As his symptoms worsened the next morning, he presented to a local emergency medical center.

**Figure 1:**
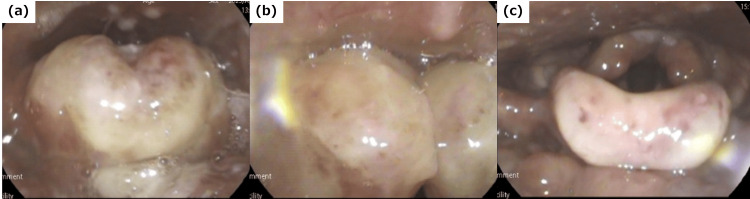
Flexible laryngoscopy (a) The epiglottis and (b) arytenoid areas were markedly swollen and reddish and had a white coating (before endotracheal intubation). (c) On the fourth day of hospitalization, the swelling had significantly subsided, with only minor pseudomembrane formation and petechiae remaining (post-extubation).

His vital signs were as follows: heart rate 98 beats/min, blood pressure 117/73 mmHg, oxygen saturation 97% (ambient air), respiratory rate 26 breaths/min, and body temperature 37.2°C. The patient was alert and exhibited no hoarseness, dysarthria, or stridor, and cervical auscultation revealed no abnormal breath sounds. Oral examination revealed a small blister on the uvula, but no other mucosal burns. Table 1 displays the results of the laboratory tests conducted during the initial presentation. Because laryngeal thermal burns were suspected based on the clinical history, flexible laryngoscopy was performed, which revealed marked swelling of the epiglottis and arytenoids with white coat formation. Given the risk of delayed airway obstruction, he was transferred to our hospital, where his edema worsened (Figure 1a, 1b). With tracheostomy on standby and with the assistance of pediatric and anesthesiology specialists, endotracheal intubation was performed under intravenous sedation using midazolam and dexmedetomidine, and the patient was subsequently admitted to the intensive care unit (ICU). Cefazolin (40 mg/kg/day) and hydrocortisone (100 mg/day) were administered for three days. By the fourth day of hospitalization, the swelling had significantly subsided, with only slight pseudomembranes and petechiae remaining (Figure 1c). He was successfully extubated following a leak test, resumed oral feeding, and was discharged on the seventh day. At the follow-up appointment on the 12th day, only minor petechiae were present, and the patient was deemed to be fully recovered.

**Table 1:** Laboratory results PT: prothrombin time; INR: international normalized ratio; APTT: activated partial thromboplastin time; AST: aspartate aminotransferase; ALT: alanine aminotransferase

Test	Results	Reference range	Interpretation
Complete blood count			
White blood cells	18.5×10³/µL	3.3-8.6×10³/µL	Elevated
Hemoglobin	13.1 g/dL	13.7-16.8 g/dL	Low
Hematocrit	40.8%	40.7-50.1%	-
Platelets	383×10³/µL	158-348×10³/µL	Elevated
Coagulation profile			
PT	13.0 seconds	10.5-13.5 seconds	-
PT (INR)	1.09	0.85-1.15	-
APTT	28.1 seconds	24.5-36.0 seconds	-
Biochemistry			
C-reactive protein	0.46 mg/dL	0.00-0.14 mg/dL	Elevated
AST	22 U/L	13-30 U/L	-
ALT	14 U/L	10-42 U/L	-
Total bilirubin	0.9 mg/dL	0.4-1.5 mg/dL	-
Urea nitrogen	17 mg/dL	8-20 mg/dL	-
Creatinine	0.46 mg/dL	0.65-1.07 mg/dL	Low
Glucose	106 mg/dL	73-109 mg/dL	-
Albumin	3.8 g/dL	4.1-5.1 g/dL	Low
Sodium	138 mEq/L	138-145 mEq/L	-
Potassium	3.8 mEq/L	3.6-4.8 mEq/L	-
Chloride	105 mEq/L	101-108 mEq/L	-

## Discussion

Thermal burns of the larynx resulting from the ingestion of overheated food or beverages are infrequent. In recent reports, only 27 cases have been documented, including 13 cases of children, with merely one case attributed to solid food [1,2]. This rarity is likely due to the immediate expulsion of hot food before it reaches the larynx [1].

Microwave ovens are widely used worldwide and can be easily operated even by children; awareness of potential hazards is essential. Caregivers and clinicians should recognize that foods heated by microwave ovens, particularly solid items, may reach dangerously high internal temperatures despite appearing safe externally. As previously described, microwave ovens heat food through dielectric heating, which depends on the polarization of molecules within the food matrix. Substances such as fats and sugars, being less polar than water, can reach temperatures substantially higher than the boiling point of water when exposed to microwave radiation, while regions with higher water content heat less efficiently [3-5]. Consequently, heterogeneous solid foods, such as rice balls, tend to develop uneven temperature distributions with localized "hot spots." These internal temperature gradients may not be detectable by touch or visual inspection, leading to the ingestion of overheated areas and subsequent oropharyngeal or laryngeal thermal injury. Clinicians should consider the possibility of thermal burns to the larynx, particularly when microwave-heated food is involved, even if oral findings are minimal and confirm the diagnosis through immediate evaluation with flexible laryngoscopy [1,4,6].

Both the onset and severity of airway edema are difficult to predict with accuracy, and the natural history of airway pathology following thermal injury remains poorly understood [7,8]. Peak edema typically develops 8-36 hours post-injury and may persist for up to four days [9-11], indicating the importance of repeated endoscopic evaluation. In line with these reports, the present case also demonstrated delayed worsening of edema more than 12 hours after the injury.

Airway management becomes crucial when obstruction is impending. In cases of pediatric thermal burns of the larynx, 12 out of 13 patients required intubation [1]. Studies from the burns literature indicate that antibiotics and corticosteroids are generally not recommended during the initial management of thermal inhalation injuries [12,13]. It remains unclear whether these recommendations are applicable to airway edema resulting from laryngeal contact burns. However, approximately two-thirds of the previously reported cases of laryngeal thermal burns received empirical treatment with intravenous corticosteroids, antibiotics, or both [1].

Empirical antibiotic use was reported in several cases, possibly reflecting clinicians' concern that mucosal injury might increase the likelihood of secondary infection. In such situations, agents with broad coverage of upper airway oral flora, such as ampicillin-sulbactam or clindamycin, would be more appropriate and have in fact been used in several cases [2,7].

Previous reports have provided no evidence that corticosteroids reduce the need for intubation, the duration of intubation, or the duration of hospital stay in laryngeal thermal burns. Although laryngeal edema leading to stridor or extubation failure can occur even in settings unrelated to thermal injury, evidence from both adult and pediatric populations indicates that prophylactic corticosteroid administration is associated with a lower risk of post-extubation airway complications, including extubation failure [14,15]. These findings suggest that, in situations where airway edema is anticipated, corticosteroid therapy may be considered as a part of airway management, although direct evidence in this specific context remains limited.

## Conclusions

Thermal burns of the larynx due to solid food ingestion in children are exceedingly rare. Microwave-heated food may generate localized hot spots that directly damage the larynx without affecting the oral cavity. Because these injuries may progress with delayed edema and cause life-threatening airway obstruction, clinicians should maintain a strong clinical suspicion and perform prompt laryngoscopic evaluation. Careful history taking, close monitoring, and timely airway interventions are essential.
